# Glycerol-based sustainably sourced resin for volumetric printing†

**DOI:** 10.1039/d3gc03607c

**Published:** 2024-01-03

**Authors:** Eduards Krumins, Joachim C. Lentz, Ben Sutcliffe, Ali Sohaib, Philippa L. Jacob, Benedetta Brugnoli, Valentina Cuzzucoli Crucitti, Robert Cavanagh, Robert Owen, Cara Moloney, Laura Ruiz-Cantu, Iolanda Francolini, Steven M. Howdle, Maxim Shusteff, Felicity R. A. J. Rose, Ricky D. Wildman, Yinfeng He, Vincenzo Taresco

**Affiliations:** a School of Chemistry, University of Nottingham Nottingham NG7 2RD UK Vincenzo.Taresco@nottingham.ac.uk; b School of Pharmacy, Nottingham Biodiscovery Institute, University of Nottingham NG7 2RD Nottingham UK; c Faculty of Engineering, University of Nottingham Nottingham NG7 2RD UK Yinfeng.He@nottingham.ac.uk; d Department of Chemistry, Sapienza University of Rome Piazzale A. Moro 5 00185 Rome Italy; e School of Medicine, University of Nottingham Biodiscovery Institute, University of Nottingham NG7 2RD UK; f Lawrence Livermore National Laboratory Livermore CA 94550 USA; g Nottingham Ningbo China Beacons of Excellence Research and Innovation Institute University of Nottingham Ningbo China Ningbo 315100 China

## Abstract

Volumetric Additive Manufacturing (VAM) represents a revolutionary advancement in the field of Additive Manufacturing, as it allows for the creation of objects in a single, cohesive process, rather than in a layer-by-layer approach. This innovative technique offers unparalleled design freedom and significantly reduces printing times. A current limitation of VAM is the availability of suitable resins with the required photoreactive chemistry and from sustainable sources. To support the application of this technology, we have developed a sustainable resin based on polyglycerol, a bioderived (*e.g.*, vegetable origin), colourless, and easily functionisable oligomer produced from glycerol. To transform polyglycerol-6 into an acrylate photo-printable resin we adopted a simple, one-step, and scalable synthesis route. Polyglycerol-6-acrylate fulfils all the necessary criteria for volumetric printing (transparency, photo-reactivity, viscosity) and was successfully used to print a variety of models with intricate geometries and good resolution. The waste resin was found to be reusable with minimal performance issues, improving resin utilisation and minimising waste material. Furthermore, by incorporating dopants such as poly(glycerol) adipate acrylate (PGA-A) and 10,12-pentacosadyinoic acid (PCDA), we demonstrated the ability to print objects with a diverse range of functionalities, including temperature sensing probes and a polyester excipient, highlighting the potential applications of these new resins.

## Abstract 2

Volumetric Additive Manufacturing (VAM) represents a revolutionary advancement in the field of Additive Manufacturing, as it allows for the creation of objects in a single, cohesive process, rather than in a layer-by-layer approach.

## Graphical abstract

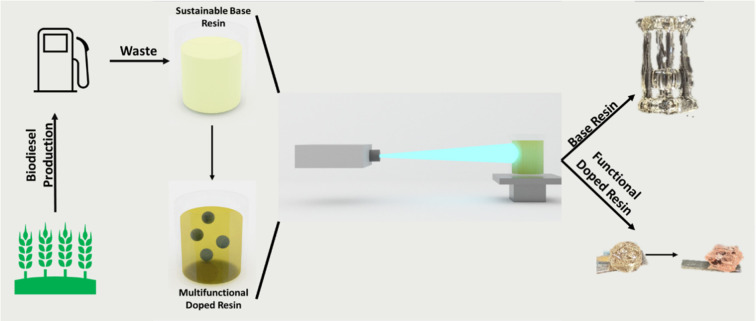

## Introduction

Additive manufacturing (AM), or 3D printing, is being widely embraced across various fields due to its capacity for innovation, efficiency, and customization. Its design freedom enables innovation and the production of complex geometries that were previously unattainable, particularly in the aerospace, healthcare, and automotive sectors.^1,2^ At the forefront of this field is the revolutionary technique of volumetric printing, which holds the promise of transforming the AM landscape. Volumetric printing or so called Volumetric Additive Manufacturing (VAM) utilises a vat polymerisation approach where a photoactive resin is rotated while a precomputed, dynamically changing light pattern is projected into the entire resin volume.^2^ The accumulation of such exposures from each azimuthal angle results in a 3D energy dose sufficient to overcome oxygen inhibition, curing the desired sections of the print. The nature of volumetric exposure enables incredibly rapid printing of macroscale objects, taking as little as one minute, while also eliminating the need for supporting structures. Consequently, it facilitates swift manufacturing and prototyping, minimising waste production.^2,3^ Although the resolution of volumetric printing can vary depending on voxelization procedures, light/project display, and the optical array used, it generally achieves a commendably high level of detail (≈80 μm), even producing thinly walled components.^3^ Two of the most significant advantages of volumetric printing over established AM techniques lie in its exceptional speed and cohesive manner in which the printed objects are formed.^2^

To meet the necessary criteria for volumetric printing, a resin must satisfy specific requirements. First, it should possess transparency, ensuring that light can pass through the liquid resin without being strongly attenuated, allowing for the creation of prints with desirable resolution.^4^ Second, the resin must exhibit photoactivity, enabling polymerisation to be triggered by incident light, thereby forming the printed object.^2^ Third, maintaining an appropriate viscosity is crucial,^2,3^ as inadequate viscosity can result in sedimentation of the printed object during the printing process, leading to complete loss of resolution. Conversely, excessive viscosity can pose challenges during sample collection and resin processing.

**Chart 1:**
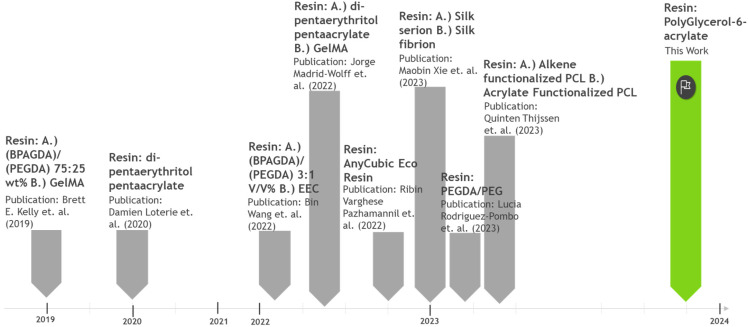
Snapshot of the nature of the resins that have been used in VAM from its inception to the present date.

Currently, the range of polymeric materials available for volumetric printing remains limited, with the majority sourced from petrochemical feedstocks. Commonly used petrochemical resins for volumetric printing include bisphenol A methacrylate, poly(ethylene glycol) diacrylate, and dipentaerythritol pentaacrylate-based resins.^3,5,6^ A very limited number of resins partially derived from renewable materials, such as gelatin-methacrylate/methcryloyl (GelMA) and Anycubic's eco resin.^7^ In the literature, polycaprolactone-based resins functionalised with alkenes or acrylates, as well as silk-based resins have also been explored.^8,9^ However, these materials face challenges related to scalability and having environmentally unsound synthesis routes. For instance, polycaprolactone based resins are produced using multi-step reactions, utilising toxic solvents and reagents such as toluene and acryloyl chloride and relatively strenuous reaction conditions are normally used (100 °C for 24 h).^9^ Clearly, there is a pressing need for resins that can be produced using robust, scalable, and environmentally conscious synthesis methodologies (Chart 1).

Glycerol, a by-product of the biodiesel and soap industries, is produced on a large scale, with production anticipated to exceed 4 billion litres in 2026.^10^ One such avenue for glycerol valorisation is through its polymerisation to form oligomeric polyglycerols (PGs). Oligomeric PGs can be synthesised by heating glycerol in the presence of an alkaline catalyst (*e.g.*, Carbonates such as Na_2_CO_3_ and K_2_CO_3_).^11,12^ Under these conditions, glycerol molecules can condense, resulting in the formation of ether linkages. The resultant product is a viscous, clear, hydrophilic polyol that has been certified as readily biodegradable. Oligomeric PGs have found application as pharmaceutical additives, humectants in personal care products, and more.^13^ In the present work, PG was identified as a promising biobased building block for volumetric printing resins due to its optical clarity, tailorable viscosity, and abundance of accessible hydroxyl groups. The availability of a platform-resin alleviates the need for in-house synthetic expertise, lowering the barrier of entry to volumetric printing.

## Materials and methods

### Materials

Polyglycerols 4,6, and 10 (PG4, PG6 and PG10) used in this study were generously donated by Spiga Nord (https://www.spiganord.com/) and are fully biobased.^14^ Acrylic acid, lithium phenyl (2,4,6-trimethylbenzoyl) phosphinate (LAP), and fluorescein (sodium salt) were purchased from Sigma Aldrich UK. 4-Methoxyphenol was purchased from Fluka. Concentrated sulphuric acid, and DMSO was purchased from Fischer Scientific. Poly(glycerol adipate) was synthesised in-house using a previously published protocol.^15,16^ 10,12-Pentacosadyinoic acid (PCDA) was purchased from Sigma Aldrich UK.

### Methods

#### Slicing the STL file for volumetric printing

A tomographic reconstruction (designed on the basis of the opensource CAL Github Repository) was used to generate the dynamically evolving projection image set for our printing system. The software takes in a 3D model (STL file) and generates a sequence of 2D grayscale images with varying pixel intensities. The software optimises the pixel intensities in such a way that when projected over a range of angles, the accumulated dose from each projection solidifies the resin when it surpasses the curing threshold. In essence, this method ensures that out-of-part sections of the resin volume do not exceed the inhibition threshold, whilst selectively curing the in-part sections of the resin volume to produce a three-dimensional structure.

#### Computed axial lithography (CAL) volumetric printer set-up

Computed Axial Lithography (CAL) is a volumetric additive manufacturing (VAM) method based on the tomographic reconstruction of a 3D light dosage distribution and was the technique used in this study^2^ (https://github.com/computed-axial-lithography). A benchtop version of a CAL system was developed based on opensource hardware design files (ESI Fig. 1†). The system was composed of a digital light processing (DLP) projector, collimating optics, and a rotary stage which carries the vial containing photocurable resin. The image sequence generated by the CAL software was sequentially projected onto the resin vial in tandem with the continuous rotation of the vial to spatially control the curing of resin within the volume. The projector used had a DMD resolution of 912 × 1140 pixels arranged in a diamond configuration and a built-in UV LED with a centre wavelength of 405 nm and a total output power of 59.6 mW cm^−2^. With 8-bit modulation capability, the projector can vary the intensity of each pixel into 256 levels allowing it to modulate light intensity throughout the projection space.

As the CAL computation assumes parallel light rays travelling through the vial, the aim of the projection optics was to create collimated light rays with enough depth of field to cover the width of the vial. Thus, a combination of doublet lenses was used to convert the diverging rays from projector to parallel beams at the vial. This resulted in a total projection size of 44 × 30 mm and a pixel size of 35 microns at the focus plane.

#### Virgin polyglycerol 6-acrylate resin

Acrylic acid was added to PG6 (3 : 1 ratio of acrylic acid to polyglycerol 6) and the mixture allowed to stir. The acrylic acid to PG-6 ratio was the molar amount based on the glycerol units in the chain. 4-Methoxyphenol (1 wt%) was added (to prevent unwanted crosslinking of acrylic acid during the esterification process acting as an inhibitor) and dissolved, before subsequent addition of sulphuric acid (2.5 wt%). Finally, the reaction mixture was heated to 60 °C and stirred at 200 rpm for 3 h. Analogous reactions were performed with polyglycerol 4 and 10. The produced acrylated PGs were used for printing purposes without any purification steps.
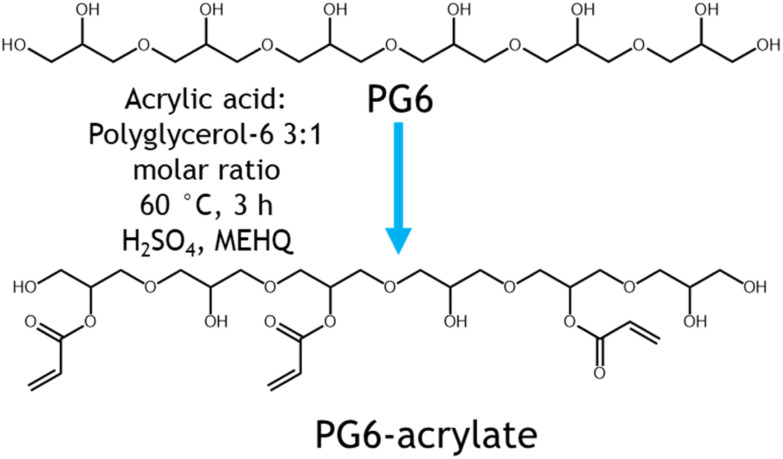


#### Rheology

Shear rate measurement experiments of the virgin resins (or free PGs) before curing were performed on an Anton Paar MCR102 rheometer, using parallel plate (25 mm) geometry at 25 °C with a measuring gap of 1 mm.

#### Poly (glycerol adipate)-acrylate dopant synthesis (PGA-A)

PGA (2.50 g, 11.66 mmol) and 4-Dimethylaminopyridine (DMAP) (0.26 g, 2.10 mmol) were dissolved in anhydrous tetrahydrofuran (THF) (15 mL). *N,N*′-Dicyclohexylcarbodiimide (DCC) (1.44 g, 6.99 mmol) was dissolved in anhydrous THF (15 mL) and acrylic acid (0.40 mL, 5.83 mmol) was added to the solution over ice. Once both solutions were homogenous, the DCC/acrylic acid solution was added to the PGA/DMAP solution dropwise over ice. The reaction was stirred over ice and allowed to warm to room temperature over 24 h. After 24 h, the reaction mixture was dissolved in ice-cold methanol and the Dicyclohexylurea (DCU) was removed by centrifugation and filtration. ^1^H NMR analysis demonstrated ≈14% functionalisation of pendant –OH groups along the polymer backbone with acrylic acid. *M*_n_ and *Đ* were determined by SEC analysis and were found to be 2670 g mol^−1^ and 2.15 respectively.

#### Polyester doped PG6-acrylate

Poly(glycerol adipate) acrylate (PGA-A) (5, 10, and 20 wt%) was added to PG6-acrylate resin and left to stir at 200 rpm for 1 h. The resulting formulation was used without any further treatment.

#### 10,12-Pentacosadyinoic acid vesicles preparation

10,12-Pentacosadyinoic acid (PCDA) vesicles were made by solubilising PCDA (4 mg) in DMSO (1 mL). The obtained solution was added dropwise to 9 mL of DI water under stirring (500 rpm) at room temperature to a final concentration of 0.4 mg mL^−1^ (1.1 mM). Then, each sample was stored at 4 °C overnight to let the system self-assemble. UV polymerization was performed at 254 nm for 20 min.

#### Temperature sensitive doped polyglycerol 6-acrylate

A suspension of 10,12-pentacosadyinoic acid (PCDA) (10, 20, and 30 wt%) was added to PG6-acrylate resin and left to stir at 200 rpm for 1 h. The resulting formulation was used without any further treatment.

#### Fluorescence imaging of printed scaffolds

Samples were prepared by slicing into approximately 2 mm cubes, transferred to a glass slide and imaged on an EVOS M5000 fluorescence microscope on DAPI (*λ*_ex_ 357/44, *λ*_em_ 447/60) and RFP (*λ*_ex_ 542/20, *λ*_em_ 593/40) filters. Subsequent image analysis was performed on ImageJ software (v 1.53) for the quantification of mean fluorescence per gel area.

#### Volumetric printing

PG6-acrylate based resin (or any doped formulation) was poured into a transparent vial. If air bubbles presented, the sample was centrifuged at 2000 rpm for 10 minutes to eliminate them from the resin. The vial was mounted onto the rotating stage and printing conditions specific for each resin and geometry were applied as specified in ESI Table 2.† Upon completion of printing, the vial was removed, and the sample collected and rinsed with deionised (DI) H_2_O to remove excess resin. The rinsed sample was placed into a UV chamber (Formlabs) and irradiated for 2 minutes to cure unreacted resin. For biocompatibility testing, samples were further processed by post-curing for an additional 60 minutes and then sequentially washed (for 60 min each) with 500 mL DI water and 500 mL 1 M aqueous solution of NaHCO_3_.

#### Dynamic mechanical analysis

A cured cylindrical sample (one for each loading formulation) (5 mm × 3.5 mm) was loaded onto the compression stage of the dynamic mechanical analyser (DMA; DMA 8000 PerkinElmer). Temperature was set to ambient room temperature and oscillation to 2 min. Storage moduli were obtained by taking the average of the data points recorded at the 2 min timepoint of the experiment.

#### Proton nuclear magnetic resonance spectroscopy

^1^H-NMR analysis of the resins was performed on a Bruker DPX 400 MHz spectrometer assigning chemical shift in parts per million (ppm). Spectra were analysed using MestreNova 6.0.2 copyright 2009 (MestreLab Research S.L.). Samples were analysed in deuterated DMSO.

#### Acid value determination

Acid value titrations were performed manually to detect the presence of free acidic groups in the virgin resin and printed scaffold. Samples were dissolved in approx. 50 mL of distilled water, and phenolphthalein (0.2 mL, 1 wt% in MeOH) was added as an indicator. The samples were titrated using aqueous sodium hydroxide (0.5 mol dm^−3^). The titration was deemed complete when the faintest pink colour persisted for longer than 15 seconds. Acid values in mg potassium hydroxide (KOH) per g sample were calculated using the equation below.
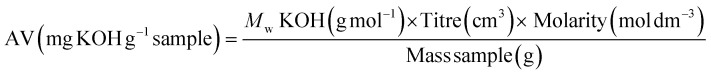


#### Raman spectroscopy

Raman spectroscopy was performed to determine the degree of curing using a Horiba Jobin Yvon LabRAM HR Raman microscope. Spectra were acquired using a 785 nm laser, a 50× objective and a 200 μm confocal pinhole. Spectra were detected using a Synapse CCD detector (1024 pixels) thermoelectrically cooled to −60 °C. Before spectra collection, the instrument was calibrated using the zero-order line and a standard Si(100) reference band at 520.7 cm^−1^. For single point measurements, spectra were acquired over a minimum range 500–2000 cm^−1^ with an acquisition time of 60 s and 2 accumulations to automatically remove the spikes due to cosmic rays and improve the signal to noise ratio. Spectra were collected from at least three random locations and averaged to give a mean spectrum.

#### Size exclusion chromatography (SEC)

Aqueous SEC was performed on an Agilent 1200 system fitted with an RI detector, Agilent two PL aquagel-OH column and one aquagel guard column eluted with 0.1 M NaNO3 eluent. Number Average Molar Mass (*M*_n_) and dispersity (*Đ*) were calculated according to polyethylene glycol (PEG) narrow standards (1500–0.105 kDa) using Agilent EasyVial calibrants fitted with a cubic function to correlate retention time and molar mass. Polymer samples were made by dissolving 2 mg mL^−1^ pure polymer in 0.1 M NaNO3. 50 μL samples were injected and eluted at 1 mL min^−1^ for 30 min.

#### Thermogravimetric analysis (TGA)

TGA was carried out using a TGA Q500 thermogravimetric analyser (TA Instruments) and 10 mg of samples for each experiment. Analysis was performed from 25 °C to 500 °C, at a heating rate of 10 °C min^−1^ under a flow of N_2_.

#### Biocompatibility

The biocompatibility of the printed samples was assessed for cytotoxicity of mammalian cells using indirect cytotoxicity testing. Before extract testing, samples (produced by breaking down in smaller specimens cylindrical printed scaffolds) were disinfected with 70% ethanol for 15 minutes then rinsed three times in sterile PBS. Extract cytotoxicity testing was performed in accordance with ISO 10993-5 methodologies. Due to the irregular shape of the printed specimens, extraction was performed at 0.2 g mL^−1^ for 24 h at 37 °C in basal culture media (BM) composed of Minimum Essential Medium Eagle (Merck, UK, Cat# M0325) with 10% (v/v) foetal bovine serum. L929 murine fibroblasts (ECACC, UK, Cat# 85011425) were seeded at 20 000 cm^−2^ in 24 well plates in 500 μL BM. After 24 h, media was exchanged for 500 μL of extract media or control media (BM incubated under the same conditions but with no extract specimen). After 24 h, negative control wells were killed with exposure to 70% (v/v) ethanol (in dH_2_O) for 15 minutes; all experimental conditions were then assayed for viability by PrestoBlue® or LIVE/DEAD™ staining.

For PrestoBlue® (ThermoFisher, UK), media (BM or extract media) was replaced with 500 μL of PrestoBlue® working solution (10% PrestoBlue® in BM) and incubated at 37 °C for 1 h. The solution was then transferred to a black 96-well plate and fluorescence detected (of the thermoresponsive dopant) at *λ*_ex_: 560 nm, *λ*_em_: 590 nm in a plate reader (Tecan Infinite 200, Switzerland), where fluorescence correlates with metabolic activity. In different wells, LIVE/DEAD™ (ThermoFisher, UK, Cat# L3224) staining was performed by removing media, rinsing the adherent cells once with PBS, adding 500 μL staining solution (2 μM Calcein AM, 4 μM Ethidium homodimer-1 in PBS) and incubating for 20 minutes at 37 °C. Cells were rinsed once with PBS, then fluorescently imaged in PBS (Leica DMI3000B). Captured images were analysed using CellProfiler (v2.2.0) by identifying and counting live (green) and dead (red) cells as primary objects (1000+ cells per condition). Samples assessed in triplicate, *n* = 3.

## Results and discussion

### Synthesis of PGs-acrylate and formulations optimisation

**Fig. 1:**
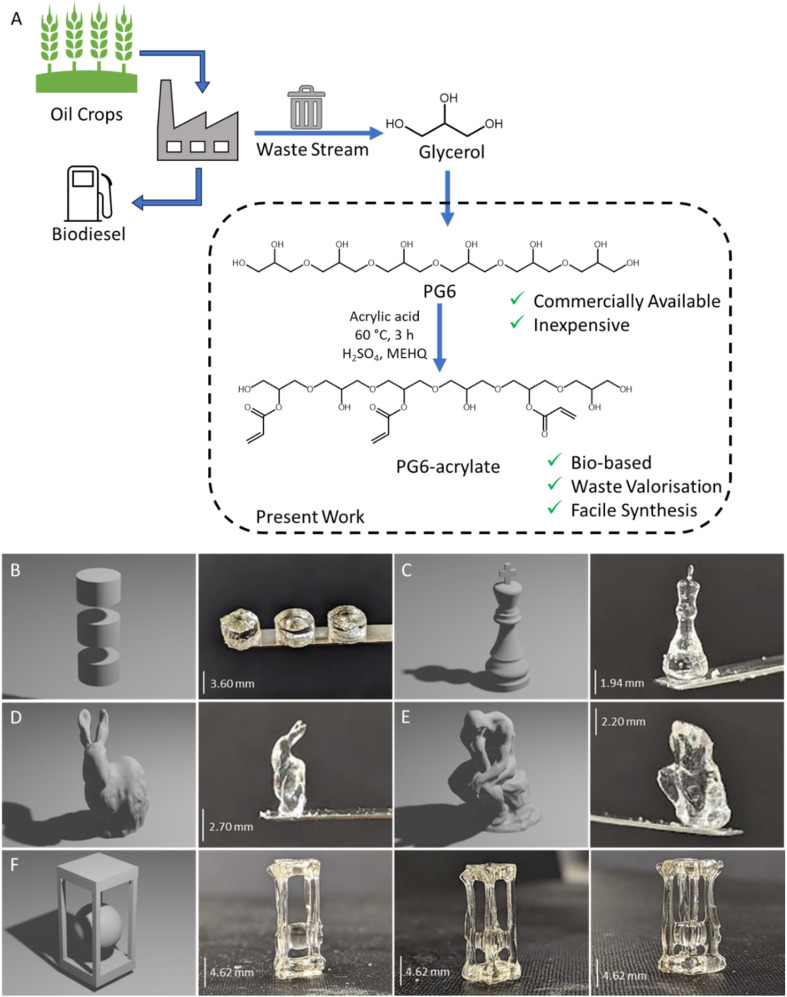
Synthesis and volumetric printing of polyglycerol acrylate-based resins. A: synthesis route towards PG6-acrylate. B–F: CAD designs and images and respective printed parts (B – cylinder (total exposure time (TET)): 6 seconds, C – chess piece TET: 6 seconds, D – rabbit TET: 18.9 seconds, E – Rodin's ‘The Thinker’ TET: 26.4 seconds, F – caged sphere TET: 18 seconds).

A sustainable polymeric resin for volumetric printing was produced through the synthesis of polyglycerol-acrylate materials. Polyglycerols were selected as the major component of the resin as they are viscous, biodegradable, transparent, biocompatible, renewably sourced, and can be functionalised with polymerisable moieties such as acrylates (Fig. 1).^17^

Direct Fischer esterification using acrylic acid and catalytic sulfuric acid was used as simple synthetic pathway to attach desired acrylate groups (to render the material photocurable) to the polyglycerol. This is a one-step route performed in bulk and under relatively mild conditions. By adopting this simple synthesis strategy, coupling reagents have been avoided, maximising atom economy and yielding a transparent polyglycerol acrylate (ESI Fig. 2†). Acrylate groups, through condensation with acrylic acid, were grafted to enable the polyglycerol to be UV curable. Acrylic acid is the simplest acrylate source and allows for the highest atom economy upon esterification with polyglycerol.^18^ Currently, acrylic acid is produced petrochemically from propene using gas phase oxidation. However, consumer demand for more responsible and sustainable processes is driving the development of biobased acrylic acid. In this context, glycerol, lactic acid, acetic acid, and furfural have all been demonstrated to be suitable biobased feedstocks to produce acrylic acid.^19,20^ As petroleum feedstocks continue to be depleted and processes are further developed, we expect that bioderived acrylic acid will become increasingly commercially competitive in the future.

Conversion of acrylic acid to PG6 acrylate was estimated to be in the region of 63% from acid value determination and 75% using ^1^H-NMR spectroscopic analysis respectively (ESI Fig. 3,4 and ESI Table 1†). This difference in conversion % could be attributed to the intrinsic differences and accuracy of the two analytical techniques adopted. However, both of them are in the same region, hinting that the majority of the acrylic acid has been attached to the PG6 backbone. In addition, PG6 showed to be thermally stable with a single weight loss step at around 300 °C while PG6 acrylate showed two additional weight loss transitions at around 100 °C (*circa* 10% of total weight) and around 200 °C (additional 20% of the total weight loss). These two steps my be due to the presence of free and bound acrylic acid plus some residual water (from catalyst and humidity) and additional reagents and catalyst degradation (ESI Figure TGA†). An increase in *M*_n_ for the functionalised PG6 acrylate was observed compared to the starting PG6 material (ESI Table SEC†). This variation in molar mass could be attributed to the different elution volume in the aqueous buffer of the acrylated polymer compared to the untreated one. Finally, PG6 acrylate presented a chromatogram with a multimodal peak likely due to the presence of different species, including unfunctionalized and mono- or multi-acylated chains, this confirming the partial functionalisation seen by ^1^H NMR and titration.

The viscosity required for volumetric printing resins resides in a “Goldilocks region”, ranging from approximately 4 to 20 Pa s, consequently, the resin viscosity was selectively tuned by altering the chain length of the PG component used. Attempts to print using PG4-acrylate were prevented by rapid sedimentation of the cured resin due to the low viscosity (ESI Fig. 5†). Conversely, PG10 was extremely viscous, making resin synthesis impractical. PG6-acrylate was found to be in the desired region, with a viscosity of 6.5 Pa s (ESI Fig. 5†) and as such was used herein.

#### Printing optimisation

Photoinitiator loading was investigated *via* the printing of cylindrical structures (Fig. 1B). LAP was selected as the photoinitiator due to its reported cytocompatibility^21^ and solubility in the PG6-acrylate resin. Different loadings between 0.05 and 1 wt% were tested, 0.075 wt% was found to be most suitable. This was chosen as higher loadings resulted in overcuring before the full frame count of the VAM process could be completed, and lower loadings were unreactive. The overcuring effect is likely to be a result of light being unable to permeate throughout the resin volume, due to excessive absorption by the photoinitiator. Lower initiator loadings (0.05 wt%) failed to produce printed parts due to insufficient polymerisation taking place in the printing process.

The performance of the resin with the optimised photoinitiator loading was thoroughly examined by printing a range of objects with diverse geometrical features. The initial focus was on printing cylindrical structures using PG6-acrylate (Fig. 1B). Once these basic structures were successfully printed, more intricate objects were fabricated, including a chess piece, a rabbit, a model of Rodin's *The Thinker* and a sphere encased within a four-pillared cage (Fig. 1C–F). It was crucial to optimise the printing conditions for each unique structure due to the voxelization program's varying intensity of projected light based on shape thickness and geometry (ESI Table 2†). If parts are under-cured the structural integrity as well as mechanical properties could be lacking, additionally if overcured the part could completely lose resolution.^6^ For example, the highest resolution for the sphere trapped in the pillared structure required 18 seconds of exposure time (under 1 min total time for the whole structure). Among the printed objects, the chess piece demonstrated the highest resolution, with the smallest feature size measuring between 300 and 500 μm (Fig. 1C).

The speed at which these parts are produced is fast when compared to other forms of vat polymerisation, *e.g.*, SLA. For comparison, cylindrical samples were printed in under 30 seconds and more complicated geometries (caged sphere) in under 1 minute (18 seconds of exposure time) (ESI Table 1†). Similar prints could take between 20 min to 1 h to print using SLA depending on the set up utilised.^22^ In contrast to layer-by-layer AM techniques, support structures are not needed in volumetric printing, as demonstrated here with prints of different size which have overhanging features that would require support. This is because the viscous resin itself acts as a support structure during printing, analogous to how unsintered polymeric particles act as a support during selective laser sintering (SLS). This showcases the suitability of PG6-acrylate resin as a sustainable material for volumetric printing.

#### Resin reusability

Reusing uncured resin in subsequent prints offers an opportunity to significantly reduce waste associated with AM but it is important that this is not done at the detriment of print quality. As residual uncured resin remains below the activation energy threshold required to overcome oxygen inhibition and initiate the polymerisation reaction, it remains a photocurable material. However, the previous low-level light exposure may have altered its composition in comparison to virgin material, *e.g.*, partial depletion of photoinitiator, necessitating modified printing parameters to achieve the same end-product.

**Fig. 2:**
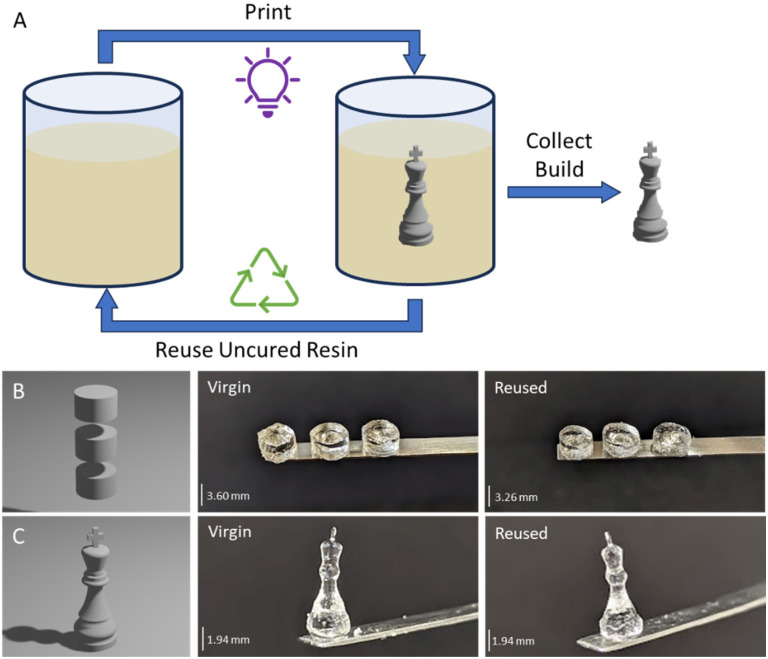
Recycling PG6-acrylate resin. A: schematic of recycling strategy. B: CAD design and images of cylindrical structures printed with virgin (left) and recycled (right) materials. C: CAD design and images of chess pieces printed with virgin (left) and recycled (right) materials.

To assess recyclability of PG6-acrylate, uncured resin was collected and used again to print an additional cylindrical structure and a chess piece (Fig. 2). It was discovered that a total exposure time of 14.4 seconds (against 12 s for virgin resin) resulted with a resolution comparable to that achieved with virgin material (Fig. 2 and ESI Table 3†). When employing the same cylindrical shape design, it was observed that the cylindrical samples printed with the recycled resin were approximately 90% of the size of those printed with virgin resin (Fig. 2B).

An additional design, with more fine features, was printed to compare the performance of the virgin resin with the recycled material (Fig. 2C). The chess pieces created from both virgin and recycled material exhibited the same base thickness and very similar minimum feature size. Notably, the recycled chess piece had a top width of 0.5 mm, while the virgin material had a width of 0.4 mm (Fig. 2C). This similarity between the virgin and recycled parts confirms the feasibility of utilising recycled material for volumetric printing.

To enhance recyclability further, it may be beneficial to combine virgin resin with a fraction of recycled resin, as is commonly practiced in other additive manufacturing techniques.^23^

#### Biocompatibility

**Fig. 3:**
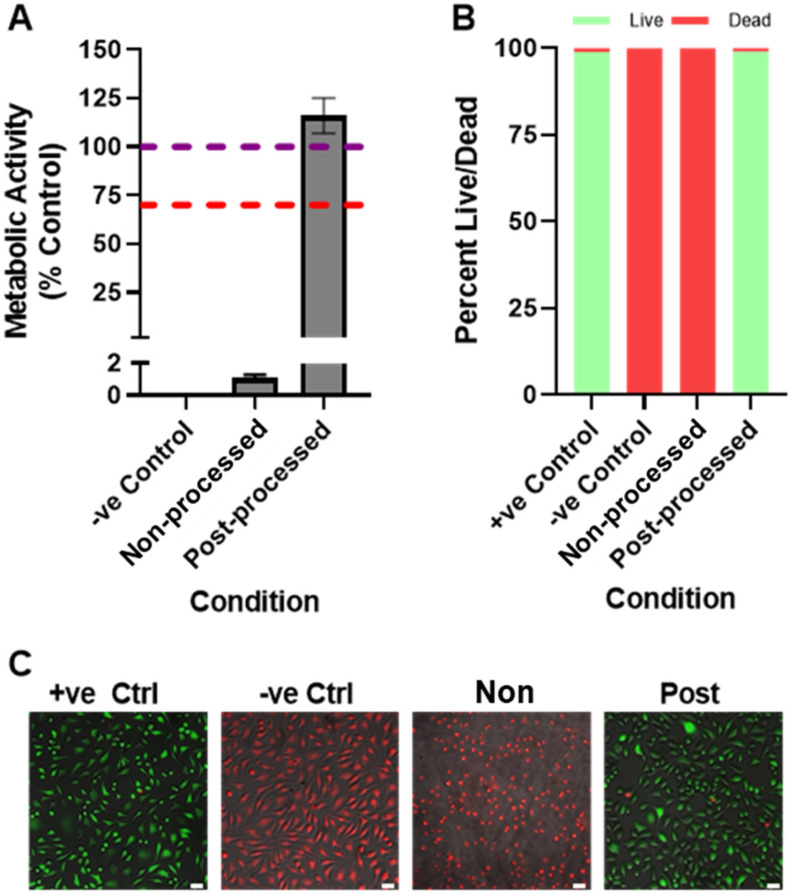
Extract cytotoxicity testing of non-processed and post-processed parts printed using PG6-acrylate using L929 fibroblasts. A: metabolic activity (purple line – positive control, red line – threshold for cytotoxicity from ISO 10993-5) (samples assessed in triplicate, *n* = 3), B: percentage of live/dead cells, C: representative images of overlaid live (green) and dead (red) images, scale bar 100 μm.

The biocompatibility of cylindrical samples printed using PG6-acrylate was assessed before and after post-processing treatments (Fig. 3).^24^ Non-processed prints made using PG6-acrylate leached cytotoxic material into the extract medium, resulting in cell death. Phenol red pH indicator present in the extraction cell culture medium turning yellow in the non-processed group indicated the presence of acidic compounds in the leachate. Micro-Raman spectroscopy was used to assess the degree of curing of the fabricated samples, measuring the ratio between signals corresponding to alkene and carbonyl-ester functionalities. The degree of curing (DC) of the post-processed PG6-acrylate build was found to be 99%, whereas a DC of 58% was measured for the non-processed build. These results suggest that the toxic leachate in non-processed samples was likely to be a combination of residual acrylic acid from the PG6-acrylate synthesis and uncured PG6-acrylate trapped within the printed polymer. Post-processing (60-minute post and aqueous washing) successfully removed these residual components by either incorporating them into the printed polymer (UV – residual PG6-acrylate) or by physical removal (washing – residual acrylic acid and PG6-acrylate), eliminating any material cytotoxicity (ESI Fig. 7†).

#### Polymeric plasticiser

In this study poly(glycerol adipate) (PGA), which is a glycerol-based polyester of biomedical significance known for its applications in drug delivery as a biodegradable carrier,^25^ was added as polymeric dopant. Poly(glycerol adipate) acrylate (PGA-A) was adopted as a second curable macromolecule and a compatible dopant (due to the presence of glycerol in its backbone) to modify the mechanical properties of the printed structures. To achieve this, PGA was partially modified introducing acrylate functionality to the secondary free hydroxyl groups *via* a Steglich esterification (ESI Scheme 1†), yielding to PGA-A (ESI Fig. 6†). This modification enables the formation of a stable 3D network between the PG6-acrylate (resin) and PGA-A (dopant) during the printing process. Importantly, despite the presence of the dopant, the resin retained its optical transparency and homogeneity, allowing the printing of cylindrical structures with a resolution comparable to that of the unmodified virigin resin (PG6-acrylate). Cylindrical structures were printed using the platform resin with varying loadings of PGA-A (0, 5 and 20 wt%, ESI Table 6†). Compression testing was performed, revealing a stark decrease in storage modulus in parts containing PGA-A. This could be attributed to PGA's low glass transition temperature <−25 °C, enabling it to act as a plasticiser at room temperature and resulting in softer parts. This outcome is highly promising and opens avenues for further exploration of readily degradable dopants to modify the end characteristics of the printed structures.

#### Thermoresponsive PCDA vesicles

**Fig. 4:**
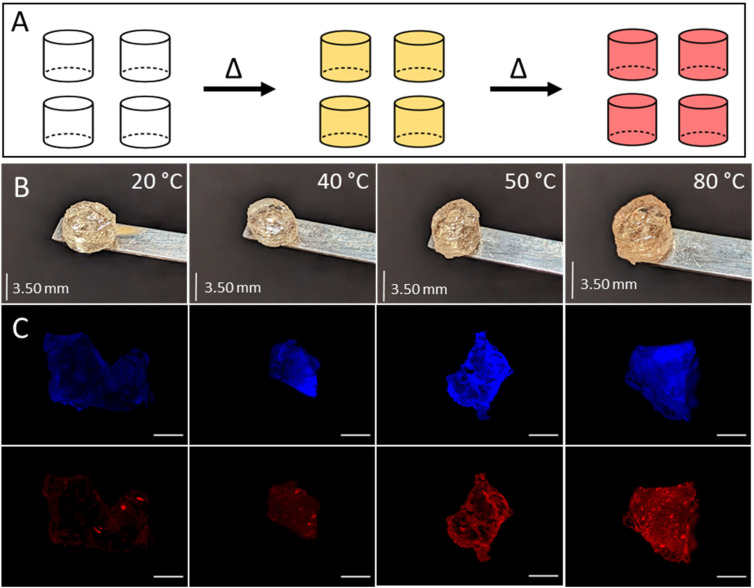
Development of a temperature sensitive resin for volumetric printing *via* ‘doping’ virigin resin with varying amounts of an aqueous suspension of 10,12-pentacosadyinoic acid (PCDA). A: schematic of 10,12-pentacosadyinoic acid (PCDA) colour change induced by temperature. B: images of printed cylindrical samples composed of polyglycerol 6 acrylate doped with 20 wt% suspension of 10,12-pentacosadyinoic acid (PCDA) after 30 seconds exposure to specific temperatures. C: fluorescent imaging of sections of printed cylindrical samples before and after 30 seconds exposure to heat. Representative images captured on DAPI (blue, top) and RFP (red, bottom) filters using an EVOS M5000 fluorescent microscope. Scale bar 500 μm.

Temperature sensing properties were incorporated into the PG6-acrylate resin by addition of micellar solutions of polydiacetylenes. When subjected to heat, polydiacetylenes undergo a transition to conjugated polymeric systems, resulting in an irreversible colour change.^26^ One example of such a thermoresponsive system is based on 10,12-pentacosadiynoic acid (PCDA).^27^ The suspensions of polydiacetylenes, both before and after thermal stimulation, exhibited narrow size distribution (ESI Fig. 8†). Specifically, the red-phase PCDA displayed larger sizes (ESI Table 7†), likely due to its conformational change from planar to nonplanar upon stimulation.^28^ To introduce thermal responsiveness into our platform resin, a suspension of PCDA crosslinked micelles was simply mixed into the resin prior to printing, highlighting the ease with which the resin can be modified. The resulting faintly blue, thermoresponsive resin was used to volumetrically print cylindrical samples. These latter resulted in a faint yellow colour (Fig. 4A and B), indicating that a partial colour change occurred during the printing process due to localised heating and a partial crosslinking within the micelles. By exposing the printed cylindrical samples to gradual increase of temperature (from room temperature to 80 °C), a colour change from faint yellow to red was achieved (Fig. 4B). This successful demonstration showcases the capability of the resin to incorporate thermoresponsive properties with a straightforward approach.

Fluorescence microscopy captured the temperature-induced change in the build in a quantitative way (Fig. 4C and ESI Fig. 9†). Before heating, the polydiacetylenes showed a weak fluorescent response, whereas after heating, fluorescence is evident and increased with temperature (Fig. 4 and ESI Fig. 9†). The colour change of the polymers can indicate the temperature in a printing vat, aiding in identifying suitable printing conditions. Additionally, thermoresponsive polymers have various applications in areas such as biomedicine, the transportation of temperature-sensitive materials (*e.g.* vaccines), and can be utilised to create wearable fever alerts without the need for thermometers.^29^

Our formulation can be used as a highly bio-sourced “platform-resin” where dopants can be introduced through simple mixing to imbue the resin with desired functionalities and behaviour. From the platform-resin, a multitude of applications could be targeted by simply altering the additives incorporated into the formulation.

## Conclusions

Our aims were to expand the range of materials available for volumetric printing and reduce our dependence to petroleum-derived resins. Innovatively, this was achieved by developing a platform based on a polyglycerol-6 acrylate resin that: (a) had the prerequisite properties needed for volumetric printing, (b) was sourced from renewable resources, (c) was produced through a sustainable, scalable, and atom economic synthetic route, and (d) could be simply modified with dopants to adapt it to a range of applications.

This platform resin could be used to print objects with a wide range of geometries, from simple cylindrical samples to complex models of *The Thinker* by Rodin. Mechanical properties could be tuned by incorporating a low T_g_ polymer, such as polyglycerol adipate acrylate, into the resin, whilst the introduction of a 10,12-pentacosadyinoic acid (PCDA) suspension as a dopant led to the development of printed parts that underwent a colour change when heated.

The transition in volumetric printing from petroleum-based resins to bio-resourced, functionalisable, and versatile systems such as polyglycerol represents an important advancement on the path towards more sustainable manufacturing. Such advancements have the potential to revolutionise not only the additive manufacturing field, but also interconnected industries such as healthcare and consumer products.

## Author contributions

Eduards Krumins: conceptualization, methodology, investigation, formal analysis, writing – original draft, review & editing; Joachim C. Lentz: conceptualization, methodology, investigation, formal analysis, writing – original draft, review & editing; Ben Sutcliffe: methodology, investigation, formal analysis, writing, review & editing; Ali Sohaib: methodology, investigation, formal analysis; Philippa L. Jacob: methodology, investigation, formal analysis; Benedetta Brugnoli: methodology, investigation, formal analysis; Valentina Cuzzucoli Crucitti: methodology, investigation, formal analysis; Robert J. Cavanagh: methodology, investigation, formal analysis; Robert Owen: methodology, investigation, formal analysis, writing; Cara Moloney: methodology, formal analysis; Laura Ruiz-Cantu: funding acquisition, review & editing; Iolanda Francolini: supervision, review & editing; Steven M. Howdle: supervision, review & editing; Maxim Shusteff: review & editing; Felicity Rose: supervision, review & editing, funding acquisition; Ricky Wildman: supervision, review & editing, funding acquisition; Yinfeng He: supervision, writing – review & editing, funding acquisition; Vincenzo Taresco: conceptualization, supervision, writing – review & editing, funding acquisition.

## Conflicts of interest

There are no conflicts of interest concerning this publication.

## Supplementary Material

GC-026-D3GC03607C-s001
