# Supplementary material for: Glycerol-based sustainably sourced resin for volumetric printing
Source: Green Chem. 2024 Jan 3;26(3):1345–55. doi: 10.1039/d3gc03607c (PMC10840650; doi:10.1039/d3gc03607c)
Supplement: GC-026-D3GC03607C-s001 [file GC-026-D3GC03607C-s001.pdf]

Electronic Supplementary Material (ESI) for Green Chemistry.
This journal is © The Royal Society of Chemistry 2024

# Supporting Information

## Glycerol-based Sustainably Sourced Resin for Volumetric Printing

Eduards Krumins,[a¥] Joachim C. Lentz,[a¥], Ben Sutcliffe,[b] Ali Sohaib,[c] Philippa L. Jacob,[a] Benedetta Brugnoli,[d] Valentina Cuzzucoli Crucitti,[c] Robert Cavanagh,[e,b] Robert Owen,[b] Cara Moloney,[e] Laura Ruiz-Cantu,[c] Iolanda Francolini,[d] Steven M Howdle,[a] Maxim Shusteff,[f] Felicity R. A. J. Rose,[b] Ricky D. Wildman,[c] Yinfeng He[c*] and Vincenzo Taresco[a*]

a. School of Chemistry, University of Nottingham, Nottingham, NG7 2RD, United Kingdom

b. School of Pharmacy, Nottingham Biodiscovery Institute, University of Nottingham, NG7 2RD, Nottingham, United Kingdom
c. Faculty of Engineering, University of Nottingham, Nottingham, NG7 2RD, UK,
d. Department of Chemistry, Sapienza University of Rome, Piazzale A. Moro 5, 00185 Rome, Italy
e. School of Medicine, University of Nottingham Biodiscovery Institute, University of Nottingham, NG7 2RD, United Kingdom
f. Lawrence Livermore National Laboratory, Livermore, CA 94550, USA
g. Nottingham Ningbo China Beacons of Excellence Research and Innovation Institute, University of Nottingham Ningbo China, Ningbo 315100, China

¥ Authors contributed equally.

* Corresponding authors: Yinfeng.He@nottingham.ac.uk, Vincenzo.Taresco@nottingham.ac.uk

## Schemes, Figures and Tables

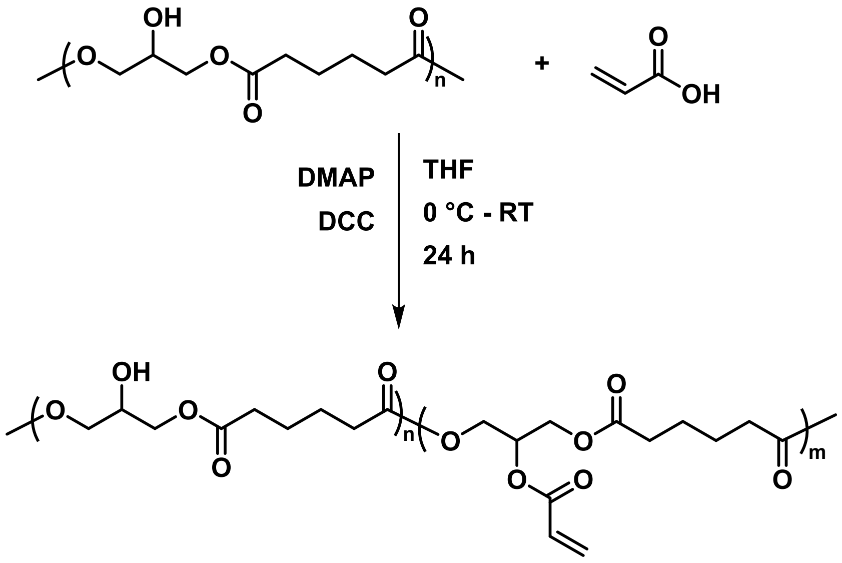


***SI Scheme 1.*** *Synthesis of PGA-A via Steglich esterification.*

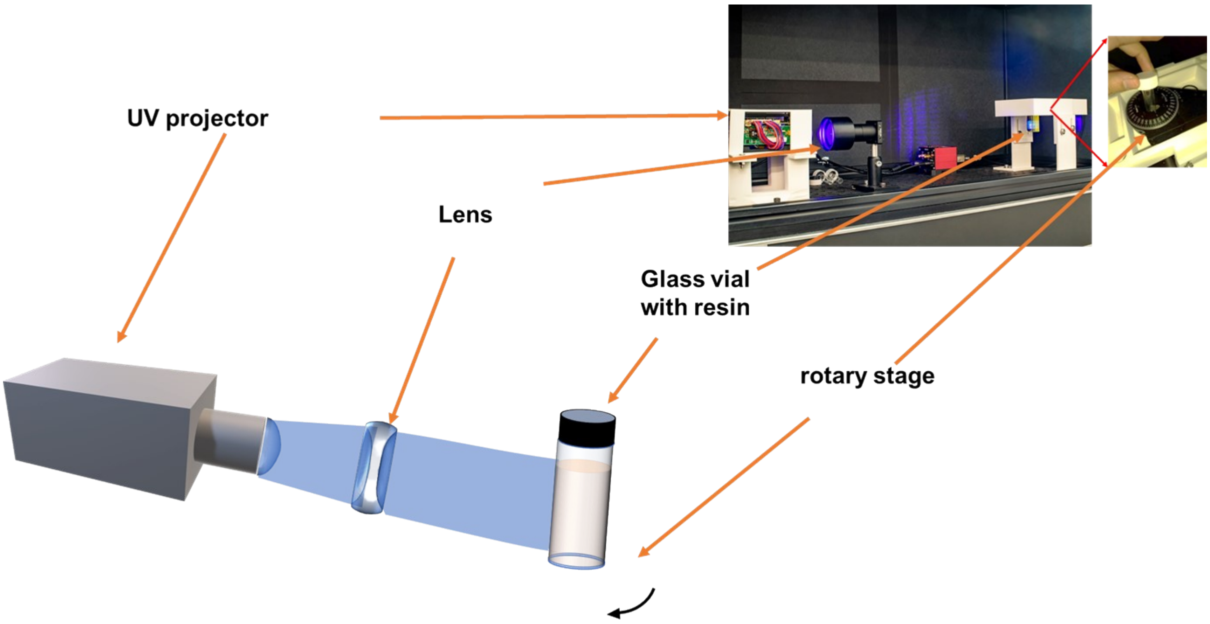


***SI Figure 1*** *Computed Axial Lithography (CAL) setup consisting of a projector, optics, and a rotary stage (based on hardware design files available from https://github.com/computed-axial-lithography).*

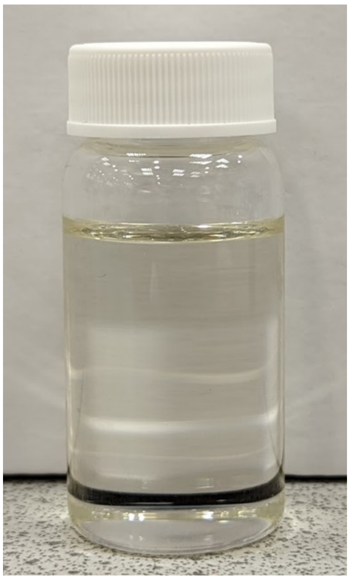

***SI Figure 2.*** *Vial containing PG6-acrylate resin.*

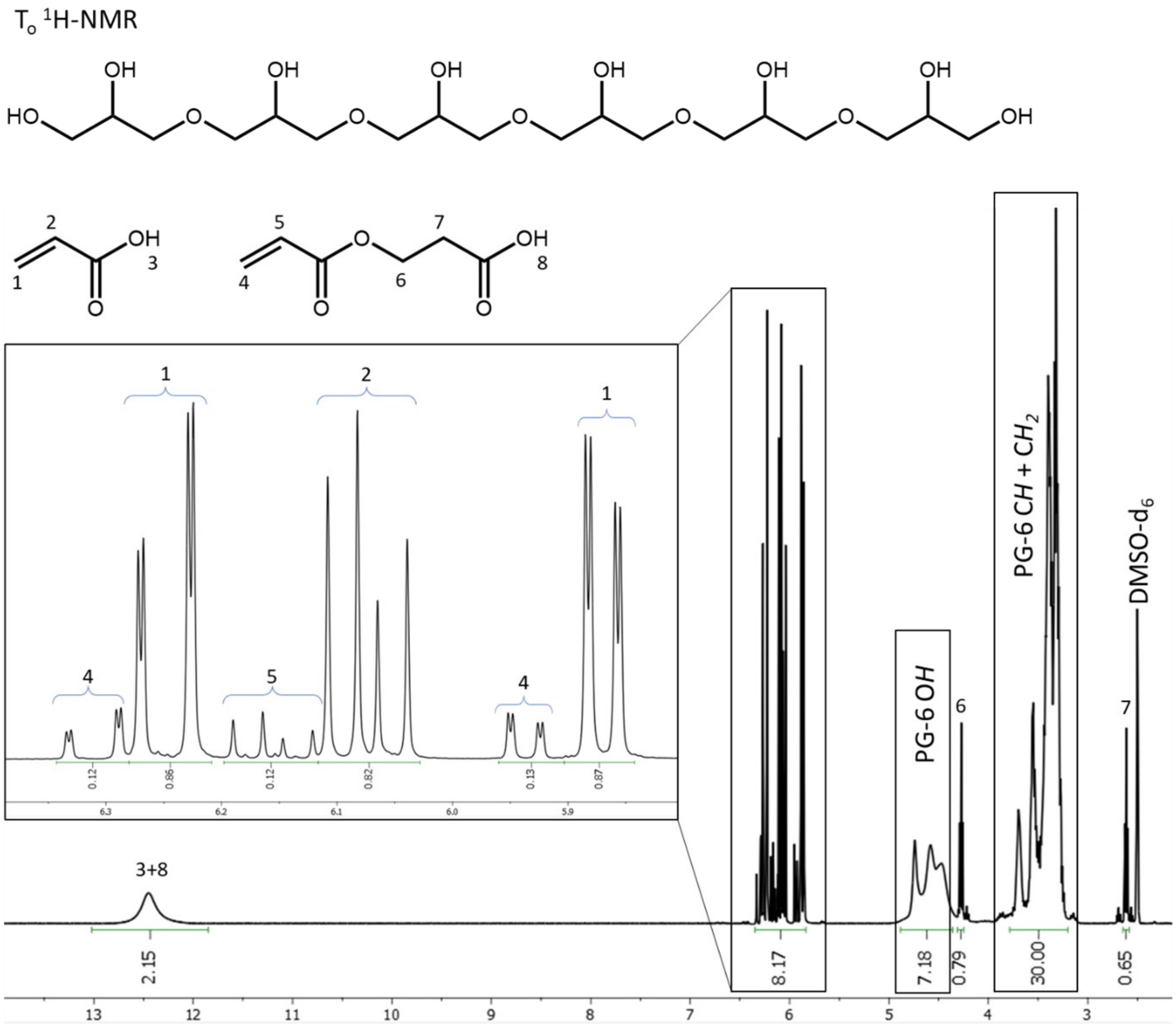


***SI Figure 3.*** *$^1$H-NMR at Time Zero of resin synthesis. Note the presence of a -COO*H *signal around 12.5 ppm, and the presence of Michael addition product in the acrylic acid starting material.*

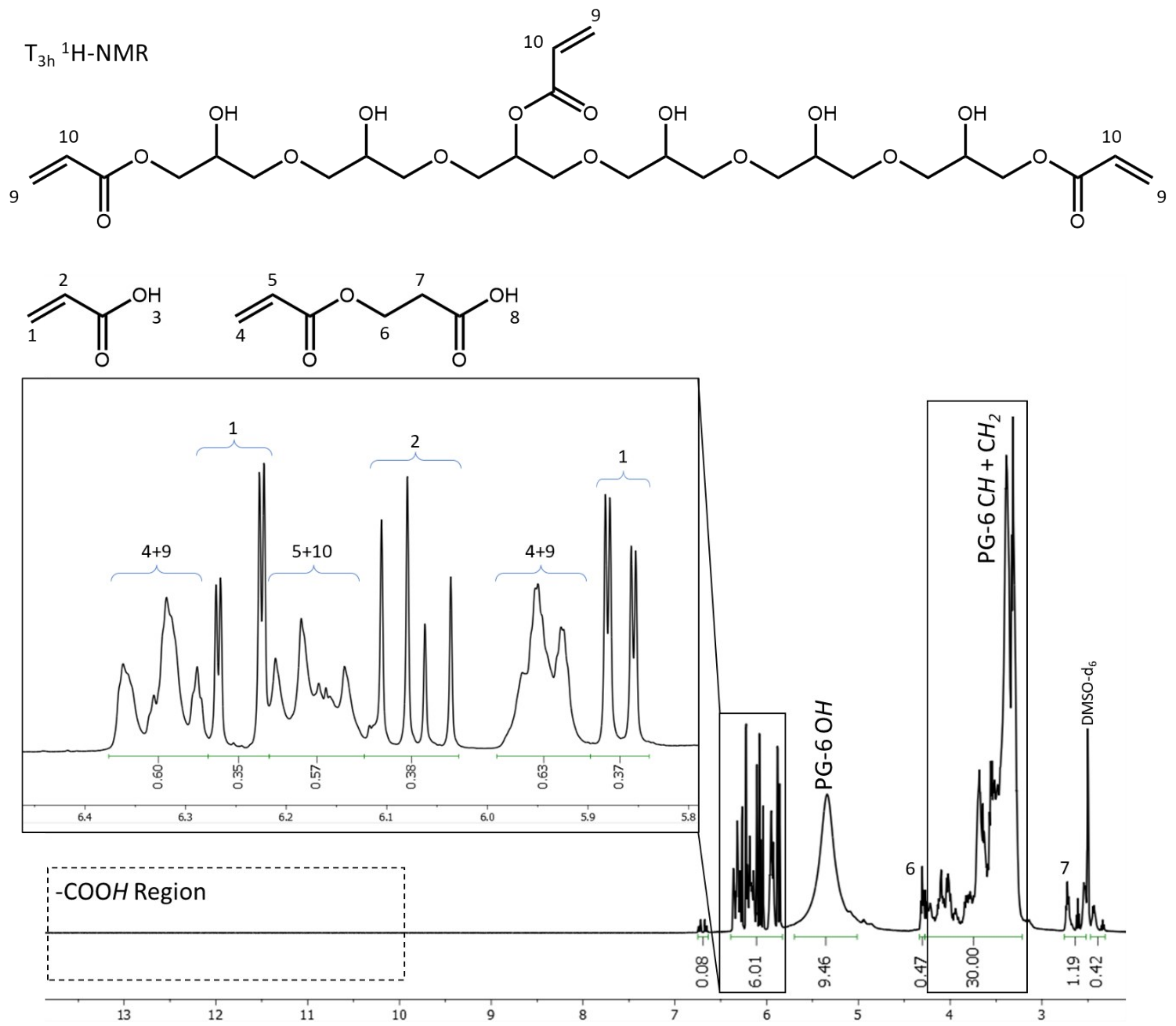


***SI Figure 4.*** *$^{1}$H-NMR at Time=3 h of resin synthesis. Inset showing the acrylate region of the spectrum. New signals corresponding to acrylate esters have formed. Conversion of acrylic acid is calculated to be approximately 63%, using the integrals of signals assigned 4+9 and 1. Some error in this estimation is a result of signal overlap between acrylic esters with the Michael addition product.*

***SI Table 1** Acid Values of the reaction mixture with correction applied to account for the presence of catalytic sulphuric acid (AV of batch used= 1112 mg KOH g$^{-1}$). Conversion was calculated following SI Equation 1.*

| Time (h) | AV (mg KOH g$^{-1}$ sample) | Conversion of Acrylic Acid (%) |
|---|---|---|
| 0 | 220.4 | 0 |
| 3 | 55.0 | 75 |

$$Conversion\ (\%) = 100 \times (1 - \frac{AV\ Time\ zero}{AV\ Time\ X})$$

***SI Equation 1** Conversion of acrylic acid (%) from acid value obtained at time zero and at measured timepoint during PG6-acrylate synthesis.*

**SI Table SEC**

| | PG4 | PG6 | PG10 | PG6 acrylate |
|---|---|---|---|---|
| $M_n$ | 250.0 | 330.0 | 1230.5 | 609.0 |
| $M_w$ | 315.0 | 467.0 | 3270.0 | 1233.0 |
| Đ | 1.25 | 1.41 | 2.65 | 2.03 |

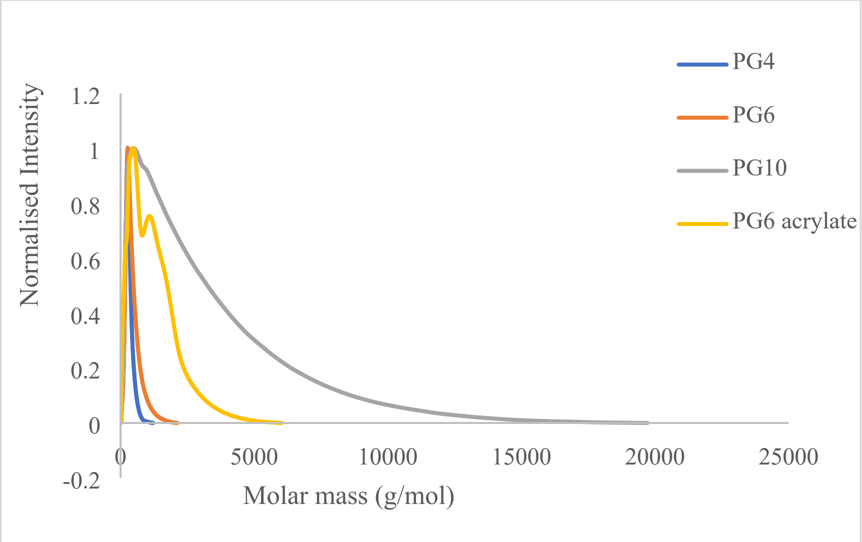


**SI Table and Figure SEC** – Molar masses (g/mol) and Đ for PG4, PG6, PG10, and PG6 acrylate. Measured via aqueous SEC.

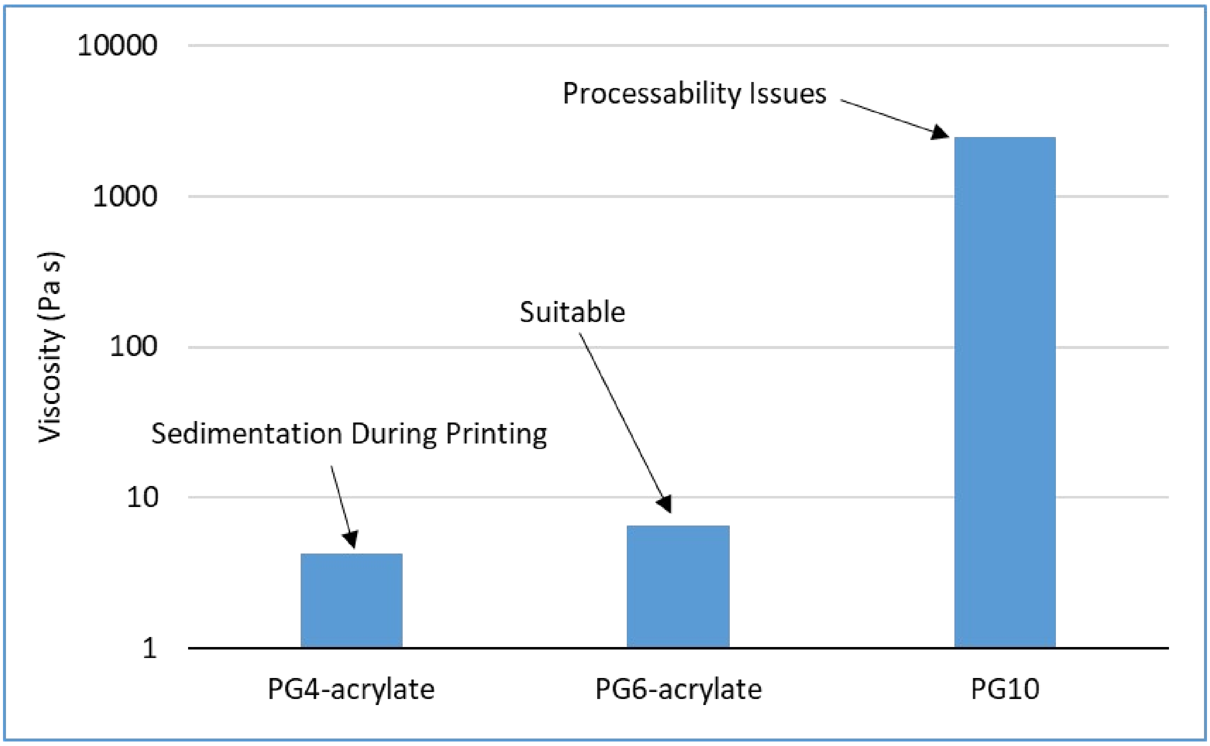


***SI Figure 5.*** *Viscosity of PG4-acrylate, PG6-acrylate, and PG10 at 25 °C, shear rate of 10 $s^{-1}$. Due to low viscosity, rapid sedimentation of the cured resin was observed with PG4-acrylate. PG10's viscosity made the resin synthesis impractical. PG6-acrylate's viscosity was found to be in the desired region.*

***SI Table 2.*** *Volumetric Printing Optimisation of virgin Resin. Print result was assessed visually based on physical appearance of the print on a scale from 1 to 10, where 1 denotes a poor print and 10 an excellent print. Parts marked with an asterisk (*) were produced using two consecutive printing cycles.*

| Part | Vial size (mL) | Exposure per frame (s) | Frames | Total Exposure Time (s) | Rotation per Frame (°) | Result |
|---|---|---|---|---|---|---|
| cylindrical structures | 4 | 0.1 | 120 | 12 | 3 | 1 |
| cylindrical structures | 4 | 0.1 | 40 | 4 | 9 | 2 |
| cylindrical structures | 4 | 0.1 | 60 | 6 | 6 | 10 |
| Chess piece | 4 | 0.1 | 60 | 6 | 6 | 10 |
| Ball in cage | 20 | 0.1 | 60 | 6 | 6 | 1 |
| Ball in cage | 20 | 0.12 | 60 | 7.2 | 6 | 2 |
| Ball in cage | 20 | 0.12 | 120 | 14.4 | 3 | 3 |
| Ball in cage | 20 | 0.12 | 210 | 25.2 | 3 | 4 |
| Ball in cage | 20 | 0.12 | 160 | 19.2 | 3 | 6 |
| Ball in cage | 20 | 0.12 | 150 | 18 | 3 | 9 |
| Rectangle | 20 | 0.1 | 60 | 6 | 3 | 10 |
| Thinker* | 20 | 0.11 + 0.11 | 120 + 120 | 26.4 | 3 | 9 |
| Rabbit* | 20 | 0.12 + 0.15 | 120 + 30 | 18.9 | 3 | 9 |
| Tensile bar | 20 | 0.12 | 60 | 7.2 | 6 | 6 |
| Tensile bar | 20 | 0.12 | 120 | 14.4 | 3 | 9 |

***SI Table 3.*** *Volumetric Printing Optimisation of Recycled Resin. Print result was assessed visually based on physical appearance of the print on a scale from 1 to 10, where 1 denotes a poor print and 10 an excellent print.*

| Part | Vial size (mL) | Exposure per frame (s) | Frames | Total Exposure Time (s) | Rotation per Frame (°) | Result |
|---|---|---|---|---|---|---|
| cylindrical structures | 20 | 0.12 | 120 | 14.4 | 3 | 10 |
| cylindrical structures | 20 | 0.12 | 140 | 16.8 | 3 | 7 |
| cylindrical structures | 20 | 0.12 | 120 | 12 | 3 | 10 |

***SI Table 4.*** *Volumetric Printing Optimisation of PGA-AA Doped Resin. Print result was assessed visually based on physical appearance of the print on a scale from 1 to 10, where 1 denotes a poor print and 10 an excellent print.*

| Part | PGA Content (wt.%) | Vial size (mL) | Exposure per frame (s) | Frames | Total Exposure Time (sec) | Rotation per Frame (°) | Result |
|---|---|---|---|---|---|---|---|
| cylindrical structures | 5 | 20 | 0.1 | 120 | 12 | 3 | 8 |
| cylindrical structures | 10 | 20 | 0.125 | 120 | 15 | 3 | 7 |
| cylindrical structures | 20 | 20 | 0.1 | 100 | 10 | 3 | 10 |

*__SI Table 5.__ Volumetric Printing Optimisation of 10,12-Pentacosadyinoic Acid (PCDA) Doped Resin. Print result was assessed visually based on physical appearance of the print on a scale from 1 to 10, where 1 denotes a poor print and 10 an excellent print.*

| Part | PCDA Content (wt.%) | Vial size (mL) | Exposure per frame (s) | Frames | Total Exposure Time (s) | Rotation per Frame (°) | Result |
|---|---|---|---|---|---|---|---|
| cylindrical structures | 10 | 4 | 0.12 | 60 | 7.2 | 6 | 5 |
| cylindrical structures | 10 | 4 | 0.12 | 80 | 9.6 | 3 | 5 |
| cylindrical structures | 10 | 4 | 0.12 | 40 | 4.8 | 6 | 8 |
| cylindrical structures | 20 | 4 | 0.12 | 40 | 4.8 | 6 | 8 |
| cylindrical structures | 30 | 4 | 0.12 | 40 | 4.8 | 6 | 3 |

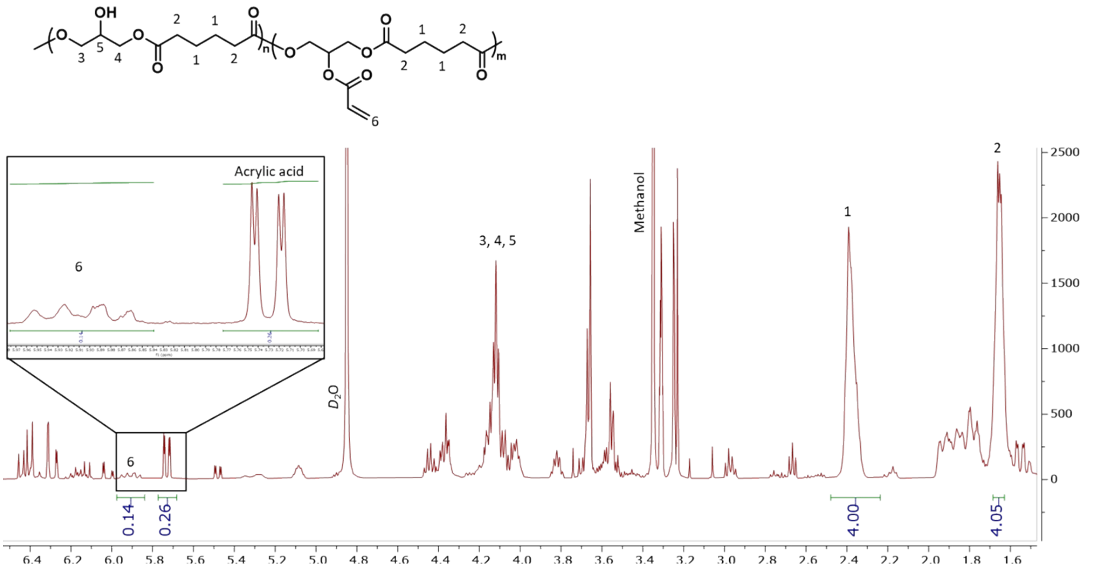


***SI Figure 6.*** *$^1$H-NMR spectrum of PGA-A in $D_2O$ demonstrating ~14% functionalisation of pendant OH bonds along polymer backbone of PGA.*

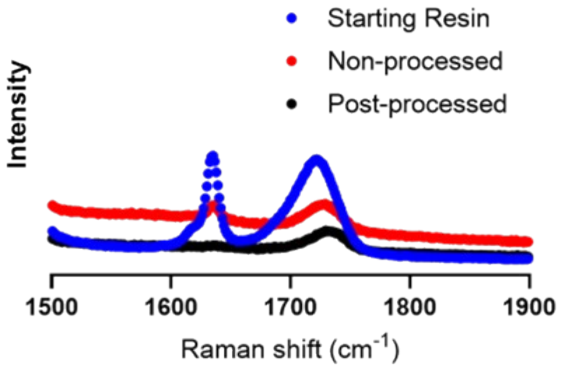


$$DC = \left[1 - \frac{(A_{c=c}/A_{C=O})\text{monomer}}{(A_{c=c}/A_{C=O})\text{polymer}}\right] \times 100$$

| Sample | DC (%) |
|---|---|
| Non-processed | 58±4.0 |
| Post-processed | 99±0.4 |

***SI Figure 7.*** *Raman Spectroscopy to assess degree of curing (DC). Left, Stacked Raman spectra of an example of starting resin, printed resin (non-processed) and resin post curing (post-processed) in the Raman shift range between 1500-1900 cm$^{-1}$. Right, DC was determined by measuring the change in the ratio of peak areas associated with the C=C (≈1634 cm$^{-1}$) and C=O (≈1720 cm$^{-1}$) bonds before and after polymerisation.*

***SI Table 6.*** *Storage modulus of printed cylindrical samples with increasing PGA-A loading.*

| PGA-A Loading (wt%) | Storage Modulus (kPa) |
|---|---|
| 0 | 2221 |
| 5 | 450 |
| 20 | 522 |

***SI Table 7.*** *Size of PCDA nanosystems non-polymerised, polymerised (blue) and after thermal stimulus (red)*

| Sample | Hydrodynamic diameter (nm) | PDI |
|---|---|---|
| PCDA | 152.1 ± 0.1 | 0.12 ± 0.10 |
| PCDA blue | 120.3 ± 0.5 | 0.21 ± 0.01 |
| PCDA red | 293.2 ± 0.8 | 0.10 ± 0.01 |

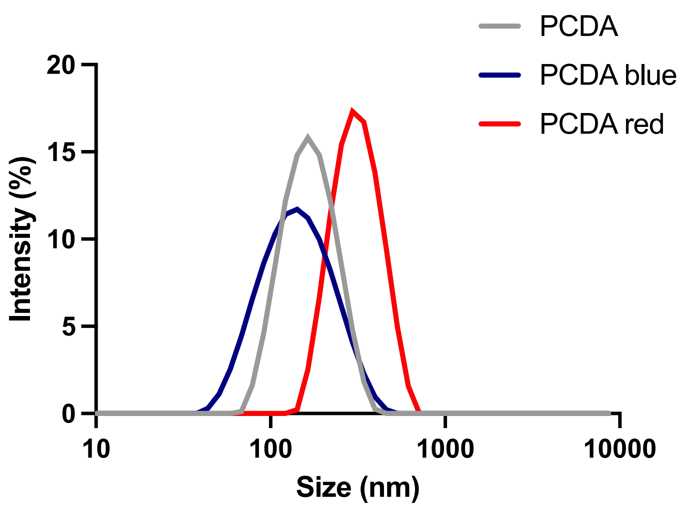


***SI Figure 8.*** *DLS traces of PCDA nanosystems non-polymerised (grey), polymerised (blue) and after thermal stimulus (red).*

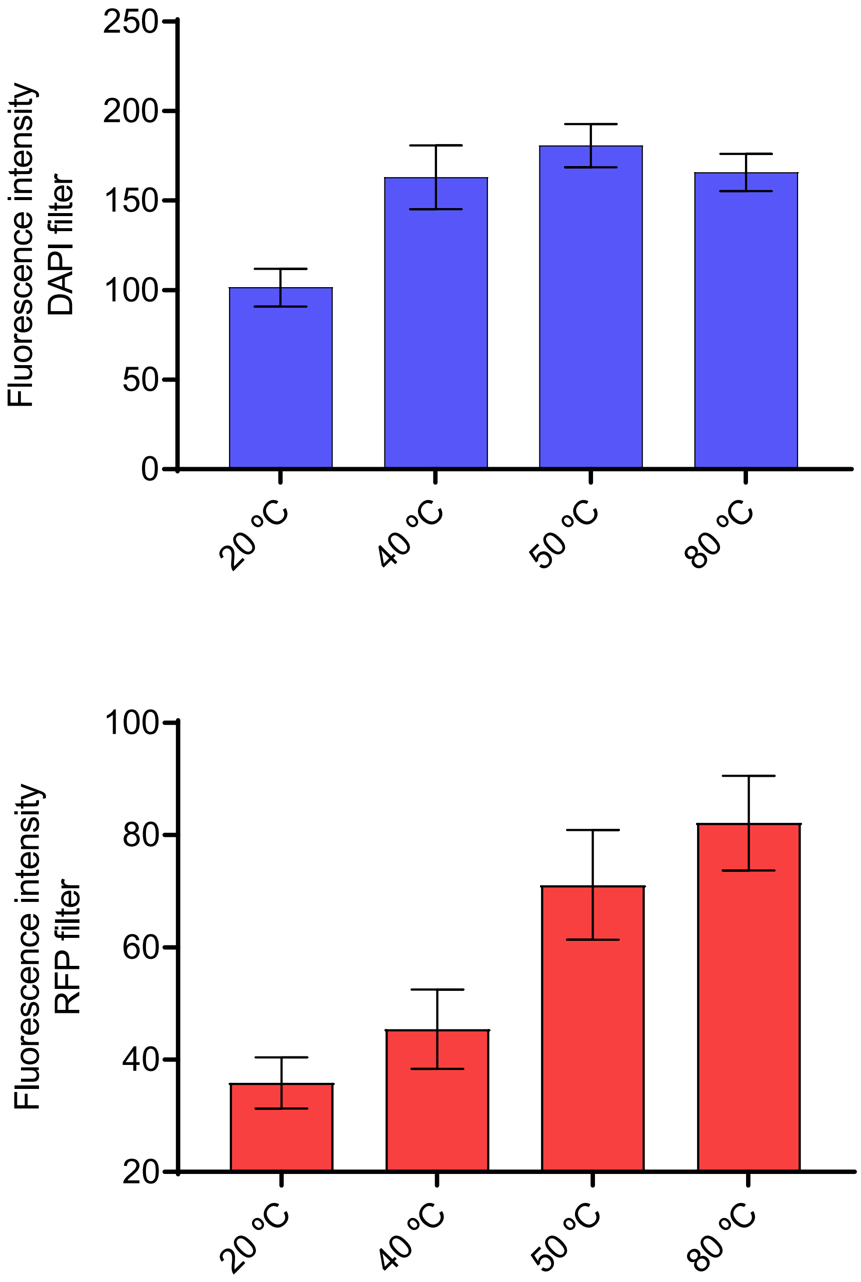


***SI Figure 9.*** *Fluorescence Intensities during microscopy, blue (top) and red (bottom) of the samples seen in Figure 4 of the main text. Data presented as mean ± S.D; n=3.*

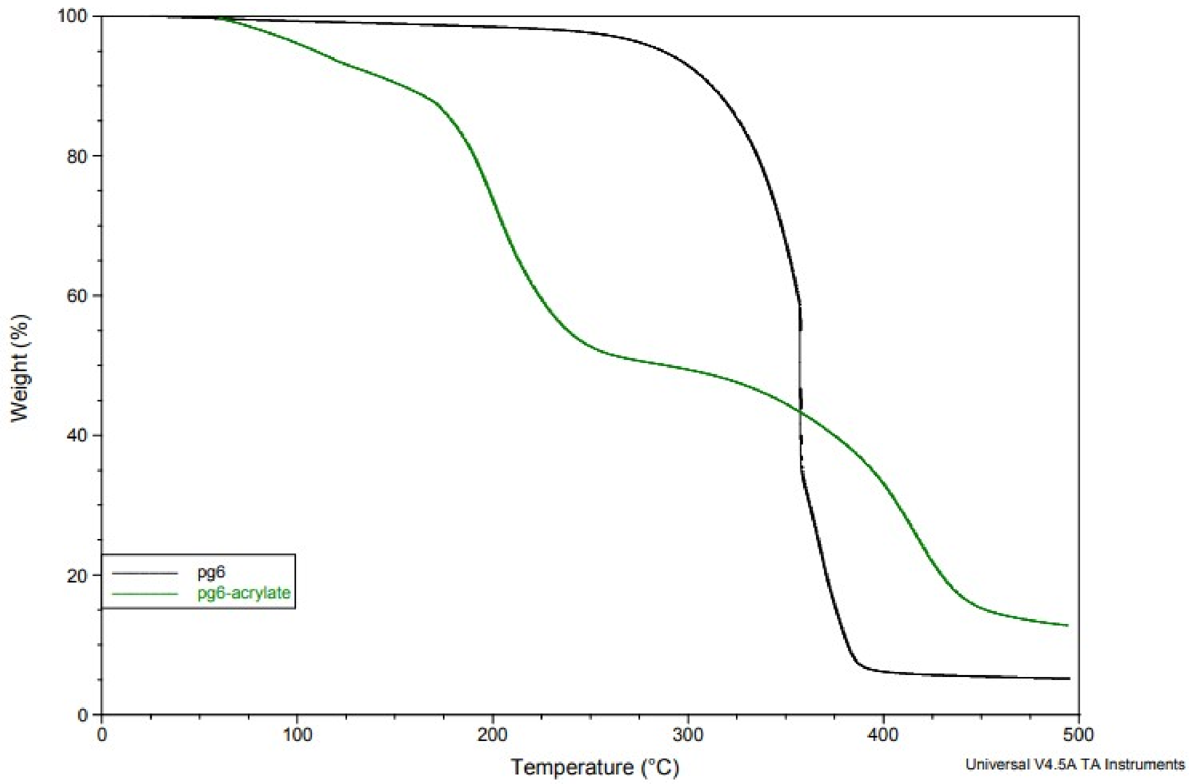

**SI Figure TGA.** TGA of PG6 and PG6 acrylate
